# Reconstructing virtual large slides can improve the accuracy and consistency of tumor bed evaluation for breast cancer after neoadjuvant therapy

**DOI:** 10.1186/s13000-022-01219-2

**Published:** 2022-04-28

**Authors:** Dandan Han, Jun Liao, Meng Zhang, Chenchen Qin, Mengxue Han, Chun Wu, Jinze Li, Jianhua Yao, Yueping Liu

**Affiliations:** 1 Department of Pathology, The Fourth Hospital of Hebei Medical University, No. 12 Jiankang Road, Shijiazhuang, 050011 Hebei China; 2 AI Lab, Tencent, Tencent Binhai Building, No. 33, Haitian Second Road, Nanshan District, Shenzhen, 518054 Guangdong China

**Keywords:** WSI Stitcher, Reconstructing virtual large slide, Efficiency and consistency, Breast cancer

## Abstract

**Background:**

To explore whether the “WSI Stitcher”, a program we developed for reconstructing virtual large slide through whole slide imaging fragments stitching, can improve the efficiency and consistency of pathologists in evaluating the tumor bed after neoadjuvant treatment of breast cancer compared with the conventional methods through stack splicing of physical slides.

**Methods:**

This study analyzed the advantages of using software-assisted methods to evaluate the tumor bed after neoadjuvant treatment of breast cancer. This new method is to use “WSI Stitcher” to stitch all the WSI fragments together to reconstruct a virtual large slide and evaluate the tumor bed with the help of the built-in ruler and tumor proportion calculation functions.

**Results:**

Compared with the conventional method, the evaluation time of the software-assisted method was shortened by 35%(*P* < 0.001). In the process of tumor bed assessment after neoadjuvant treatment of breast cancer, the software-assisted method has higher intraclass correlation coefficient when measuring the length (0.994 versus 0.934), width (0.992 versus 0.927), percentage of residual tumor cells (0.947 versus 0.878), percentage of carcinoma in situ (0.983 versus 0.881) and RCB index(0.997 versus 0.772). The software-assisted method has higher kappa values when evaluating tumor staging(0.901 versus 0.687) and RCB grading (0.963 versus 0.857).

**Conclusion:**

The “WSI Stitcher” is an effective tool to help pathologists with the assessment of breast cancer after neoadjuvant treatment.

## Background

Neoadjuvant therapy (NAT), the treatment given as a first step to shrink and downstage the tumor before the main treatment, has been established as an effective practice in the overall treatment for selected breast cancer patients. NAT can make inoperable breast cancer patients for surgical resection or make some patients for breast-conserving surgery instead of mastectomy [1, 2]. Furthermore, NAT allows the evaluation of tumor sensitivity to drugs and guides subsequent adjuvant treatments [3]. The pathologic evaluation of post-NAT breast specimens not only allows the assessment of therapeutic efficacy but also enables the prediction of patient prognosis [4–7]. Pathologic complete response (pCR) after NAT can also be used as an alternative endpoint to prognosis in clinical trials of neoadjuvant drugs for breast cancer. Therefore, accurate pathologic evaluation of post-NAT breast cancer is crucial for the prescription of the patient’s treatment regimen and prognostic prediction. The Breast International Group-North American Breast Cancer Group (BIG-NABCG) strongly recommends using the Residual Cancer Burden (RCB) index to evaluate patients with post-NAT breast cancer [8].

Conventionally, to calculate the RCB, pathologists delineate the tumor margin with marker pen under the microscope and then stack splicing the glass slides together to restore the tumor for measurements. However, in actual practice, many factors can influence the tumor measurement and RCB calculation, such as the difficulty of stack splicing an excessive number of slides, tissue deformation after it is made into pathology slides, delineation errors under the microscope, pathologist’s familiarity with the procedures and pathologist’s error in the repeated measurement of the same case [9, 10]. In particular, stack splicing the physical glass slides is inconvenient. Holding the stack stable is more challenging when there are over five tissue fragments. The restored tumor cannot be saved in a high-resolution digital form for future analysis as well. On the other hand, the stack splicing procedure is a very subjective process that the splicing results between different physicians may vary to each other, which can affect the accuracy of tumor size measurement thus lead to inaccurate RCB index.

However, with the popularization of whole slide imaging (WSI), a technology that translates a conventional glass slide into a digital form, some pathologists attempt to evaluate the restored tumor bed through digital WSI fragments stitching [11]. For example, the HistoStitcher [12] program can facilitate WSI fragments stitching by defining pairs of matching points between two adjacent tissue fragments. The same iteration is repeated until the stitching is completed. However, the stitching process is still not efficient since it requires the user to carefully examine the cut between sections to identify good matching points which are the key to successful stitching. Multiple attempts are often required to ensure a good stitching outcome. Penzias et al. [13]also developed a stitching software for WSI fragment stitching. This program can extract the tissue foreground from the background of the digital slide, while also detecting the contour and extracting the edge of the cut, to achieve gap compensation and stitching between tissue fragments. However, this approach is only suitable for slides with regular matrix-based distribution (e.g. a 2*2 distribution) and requires clean and neat edges. Therefore, it may fail when the reconstructed tumor has irregular spatial distribution. In addition, since linear fitting is used for gap compensation at the cutting edges, stitching at edge areas is more error-prone. Benoît et al. [14] proposed a method of using low-resolution thumbnails to perform WSI fragment stitching by calculating the correlation coefficients of adjacent slides. The advantage of this method is that the size of the thumbnails is very small, and the stitching process has lower requirements for computer hardware. However, this correlation coefficients-based method can go wrong when the adjacent WSI fragments have gaps at the cut edge, which is quite common due to the imperfect pathological slicing process.

In this study, we explore a more effective and accurate method of virtual large section reconstruction, which aims to improve the accuracy and efficiency of tumor bed evaluation in post-NAT breast cancer. Virtual large slides(VLS) are useful for the evaluation of large tumors that require multiple pathological slides, such as prostate cancer [15] and breast cancer. We developed a WSI fragment stitching software–“WSI Stitcher”. This software allows pathologists to rapidly and accurately reconstruct the full appearance of the tumor bed in post-NAT breast cancer and provides a flexible stitching mode without restrictions in spatial distribution regularity and the length and area measurement functions to obtain an accurate RCB index.

In addition, the WSI Stitcher can save the reconstructed VLSs as TIFF format with a lossless resolution, which can be opened by Openslide [16] and used for future image analysis including artificial intelligence-assisted interpretation of indicators such as tumor-infiltrating lymphocytes (TILs). This is of crucial significance for the accurate and comprehensive evaluation of post-NAT breast cancer.

## Methods

### Study cohort

**Fig. 1:**
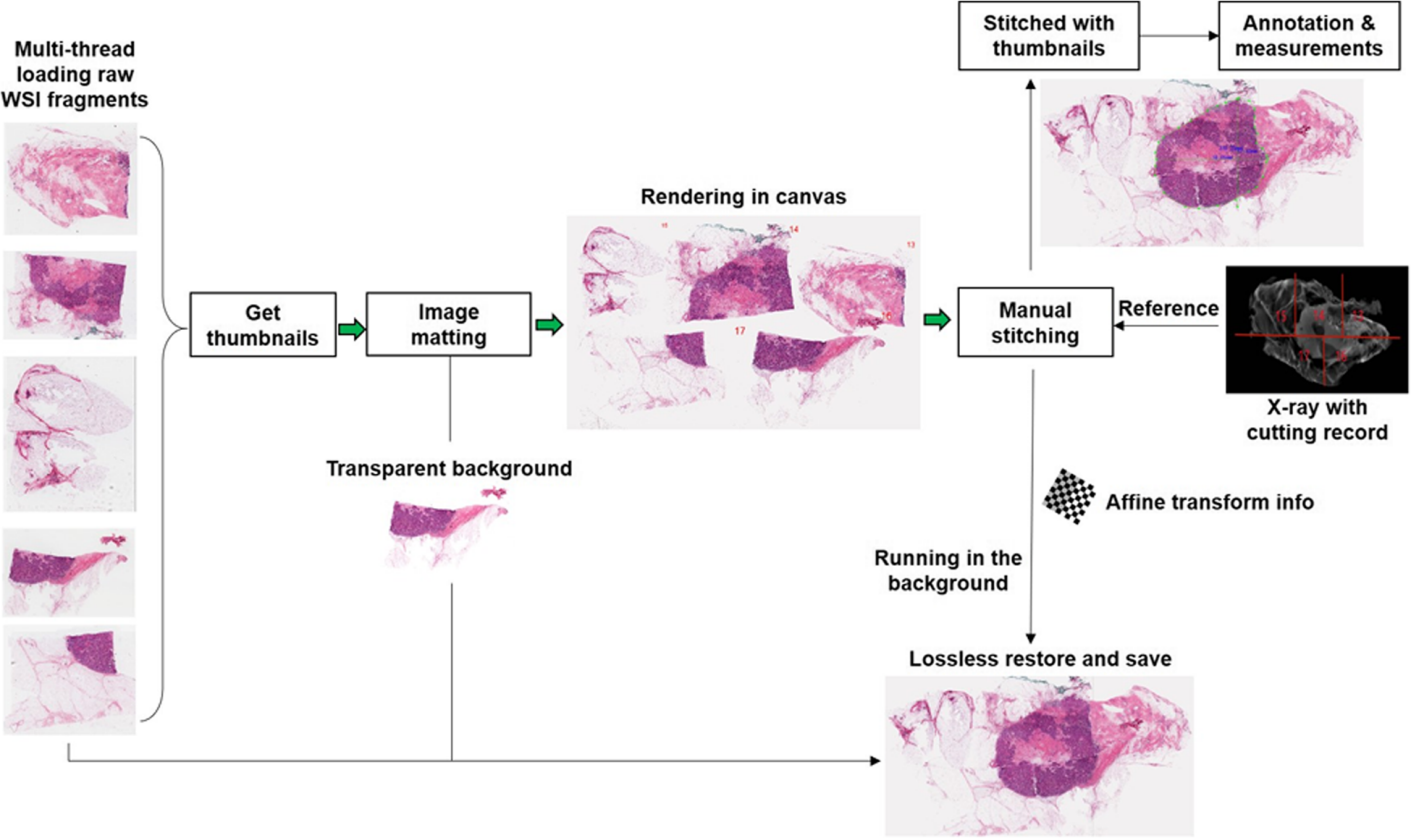
The workflow of “WSI Stitcher”. Thumbnail images of WSI fragments which are downsampled by 64 folds are imported for stitching. Image matting is performed to make the image background transparent to avoid occlusion between images during the stitching process. An X-ray image with cutting records is used to assist rapid stitching. Once the stitching is completed, the lossless reconstruction starts running in the background according to the affine transform information, which does not affect the annotation and measurements of the current specimen or the stitching of the next specimen

Specimens were collected from 80 patients who underwent radical mastectomy for breast cancer after NAT between April 2020 and February 2021 at the Fourth Hospital of Hebei Medical University. The gross tumor location was determined based on the titanium clips, skin texture localization, and preoperative imaging data. Multiple cross-sections were made at 0.5-1 cm intervals to expose the breast and tumor tissue. All tumor cross-sections were imaged using a Cabinet X-ray system (CXS) [17](Biovision Inc.), and the largest cross-sectional area of the residual tumor bed was selected to measure the maximum diameters of the largest section of the tumor bed (length and width). The specimens were then photographed and archived in the cutting direction. The largest cross-sectional area of the tumor was sampled sequentially (the sampling of marginal tissue was maximized to ensure the integrity of the largest two-dimensional area) and was placed in cassettes of a specific color to ensure that a single section corresponded to one serial number. The cutting paths were recorded and labeled on the X-ray image to assist the stitching of WSI fragments as shown in Fig. 1.

Tumor staging was assessed according to the eighth edition of the AJCC.Tumor staging was performed according to the size of the tumor bed, which was divided into T1 (≤20 mm), T2 (20-50mm), T3 (> 50 mm), and T4 (any tumor size, involving the chest wall or skin) [18]. Patients with T4 stage were excluded because T4 stage was not associated with tumor size assessed in this study.The RCB score measures the length and width of the tumor bed under a microscope, evaluates the percentage of residual tumor cells in the tumor bed and the proportion of carcinoma in situ, determines the number of lymph node metastases and the largest diameter of lymph node metastases, calculates the RCB score, and determines the RCB Grading(RCB = 1.4 (invasive cancer Percentage × tumor bed tumor size) ^0.17^+ [4 (1–0. 75 ^number of metastatic lymph nodes^) × maximum diameter of lymph node metastasis] ^0.17^).The RCB score is divided into grades 0 to III [19]. Grade 0 has the same meaning as pCR, indicating that the infiltrating lesions have achieved complete remission; RCB score > 0, ≤ 1.36 is grade I, showing a small amount of residual lesions, and partial remission of infiltrating lesions;RCB score > 1.36, ≤ 3.28 is grade II, moderate residual lesions can be seen, and infiltrating lesions are partially relieved;RCB score > 3.28 is grade III, and extensive residual lesions can be seen [20].

### WSI Stitcher software

WSI Stitcher is a WSI fragment stitching software developed in our group. It can perform import, rotation, mirroring, movement of multiple WSI images, and stitching of WSI fragments to restore the VLS. In addition, WSI Stitcher also provides flexible length and area measurement, labeling functions, and can automatically calculate the percentage of residual tumor cells in the tumor bed and the percentage of carcinoma in situ according to the user’s labels. The programming language of WSI Stitcher is Python, and the main toolkits used are Openslide [16] and PyQt4 [21]. Openslide is a C language library that provides a simple interface to read WSIs. PyQt4 is a toolkit for creating GUI applications and is used to write the application interface of python programs.

For rapid image import and stitching, as shown in Fig. 1, we first use the WSI stitcher to import the thumbnails of WSI fragments, downsampled by 64 folds, for stitching. We make the image background transparent through image matting. This can avoid mutual occlusion between WSI fragments during the stitching process. The user starts stitching by referring to a prescanned X-ray image with the cut edge record. Once the stitching is completed and confirmed by the user, the affine transform information is recorded for the lossless reconstruction of the virtual large slide. This reconstruction process runs in the background without interrupting the doctor’s workflow. All WSI stitching experiments in this paper were done on a ThinkPad T470 laptop configured with Window10 Pro, 8G RAM, 256G SSD storage, and Intel i5 CPU.

### Conventional and software-assisted evaluation methods

Five pathologists (A-E) were asked to perform the conventional evaluation and “WSI Stitcher” assisted (software-assisted) evaluation of the tumor bed in post-NAT breast cancer specimens, and to record the operation time. There is a 2-week forgetting period between the two procedures.

**Fig. 2:**
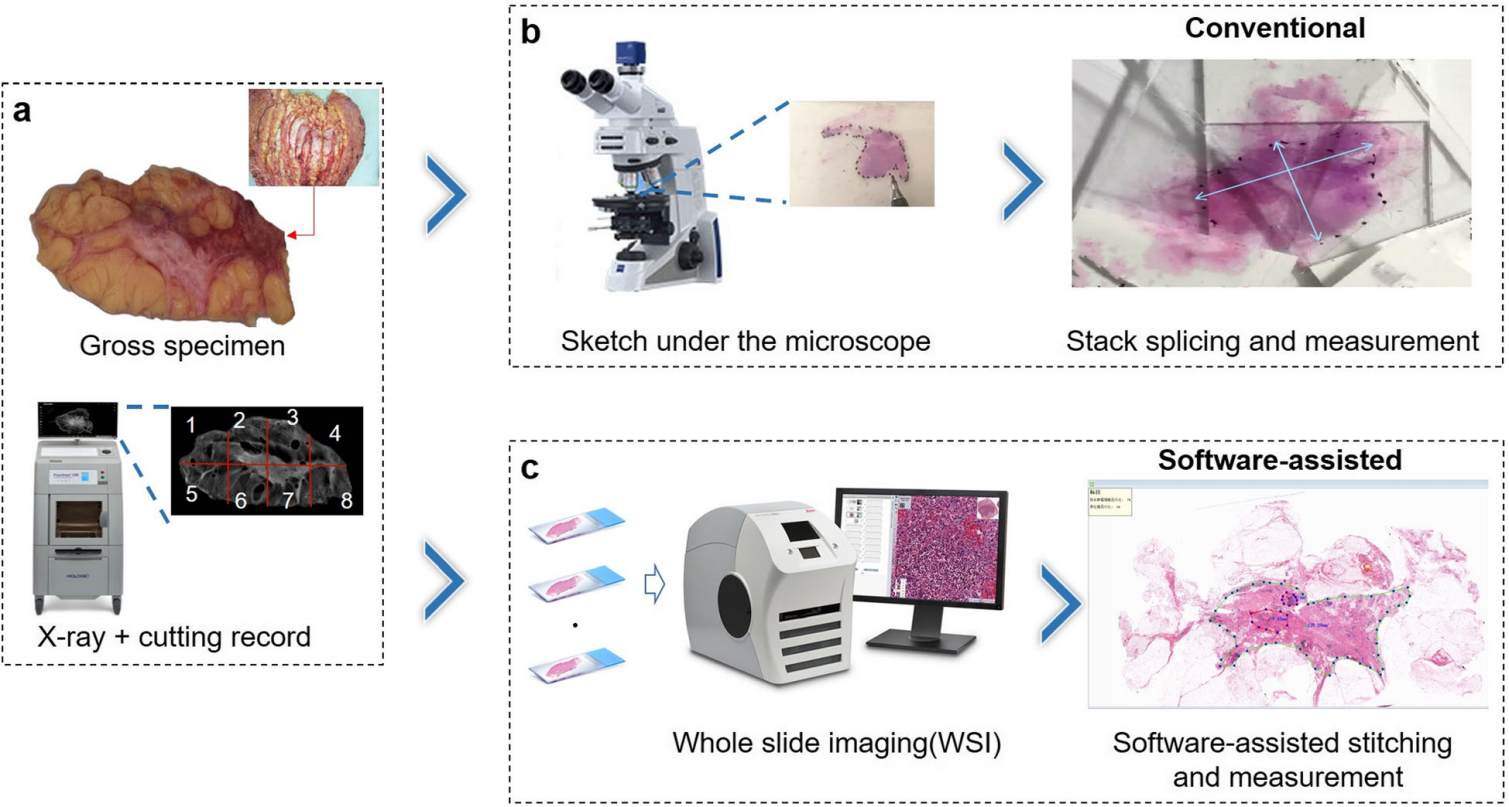
Flowchart of conventional and software-assisted tumor bed evaluation methods. **a** Take the regular color image and an X-ray image of the largest residual tumor bed and record the cutting positions. **b** conventional evaluation method: Draw the edge of the tumor under the microscope, manually stack splice the pathological slides according to the cutting record, and assess the tumor bed. **c** software-assisted evaluation method**:** Scan H&E slides to generate WSI images, and then import them into WSI Stitcher for stitching. The software has built-in length and area measurement functions. The percentage of residual tumor cells in the tumor bed and the percentage of carcinoma in situ will be automatically calculated and displayed in the upper left corner once the labeling is completed

The flow of the entire experiment is shown in Fig.2. First, the regular color image and the X-ray image of the largest section of the tumor are taken as shown in Fig. 2a. This section is then cut into several pieces to make hematoxylin-eosin(H&E) slides. The cutting positions are recorded to assist the future stitching of WSI fragments. Once the H&E slides are ready, we use two methods to reconstruct and evaluate the tumor bed respectively as shown in Fig. 2b and c. The conventional tumor bed evaluation method, as Fig. 2b shows, was performed as follows: First, the tumor edge of each glass slide was delineated individually under a microscope, then the glass slides were stacked splicing by referring to the cutting records, and the maximum diameter of the tumor bed was measured. Finally, the percentage of residual tumor cells in the tumor bed and the percentage of carcinoma in situ were evaluated.

On the other hand, the software-assisted evaluation method, as shown in Fig. 2c, was performed as follows: First, all H&E slides of tumor bed specimens collected from the post-NAT breast cancer patients were scanned to produce WSI images and then imported to WSI Stitcher for stitching. With the help of the pre-scanned X-ray of the sample and the cut edge label, pathologists performed operations such as rotating, flipping, and moving on multiple WSI images to stitch the digital slides into a VLS, to reconstruct the full appearance of the tumor bed. Furthermore, our software offers flexible functions, such as length and percentage measurement, which can assist pathologists in accurately interpreting the findings. The pathologists were also able to use different colors to label the tumor bed, and regions of invasive carcinoma and carcinoma in situ(CIS), while the percentage of residual tumor cells and the CIS in the tumor bed were automatically displayed in the top left corner. These features can simplify the process of tumor bed evaluation in post-NAT breast cancer and obtain more accurate evaluation results.

### Statistical analysis

All the statistical results were analyzed using SPSS 26.0 (SPSS Inc., Chicago, IL, USA) statistical software. The intraclass correlation coefficient (ICC) was used to analyze the consistency of the pathologists’ interpretation results using the conventional evaluation method, and that using the software-assisted evaluation method. The Fleiss’kappa (FKS) was applied for concordance analysis on the RCB grade and tumor staging.Paired samples t-test was performed to compare the average time needed for post-NAT tumor bed evaluation between the two methods. Diagrams were plotted using GraphPad PRISM 8.0. *P* < 0.05 was considered statistically significant.

## Results

### Examples of conventional and software-assisted tissue fragments stitching

**Fig. 3:**
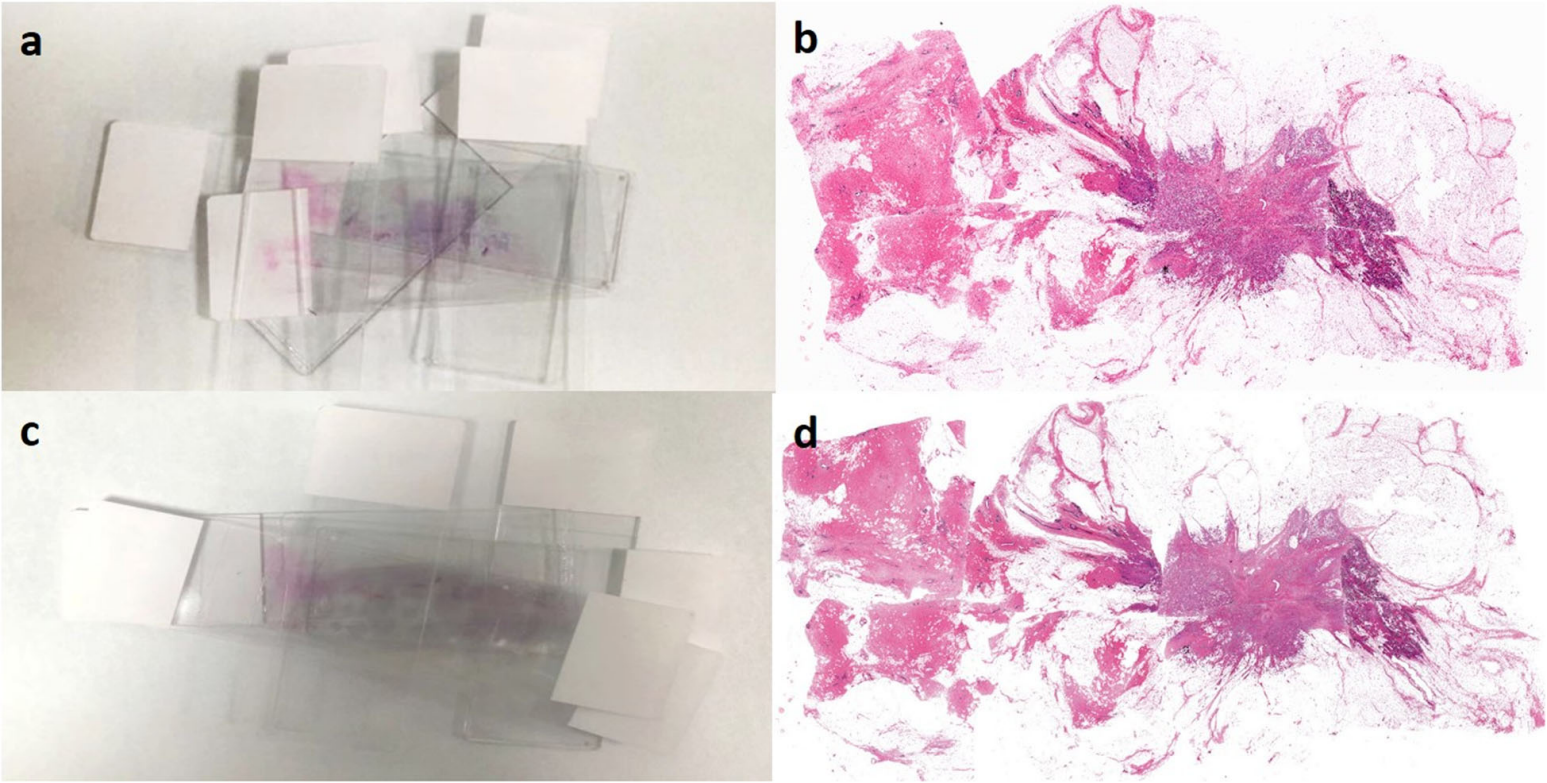
Case demonstration of conventional manual stack splicing and software-assisted stitching. **a** and **c** are the largest sections of the tumor bed obtained by two pathologists (A and B) through conventional stack splicing methods, respectively. **b** and **d** are the virtual large slides obtained by pathologists A and B using software-assisted stitching methods, respectively

As shown in Fig. 3a and c, manually superimposed slides using conventional stack splicing methods overlay each other and display incompletely. Furthermore, different pathologists often outlined the edges of the tumor bed differently, resulting in different spliced pictures and errors in the measured values. Figure 3a and c respectively showed the results of two pathologists using conventional methods to stitch the same case. The results show that the two manual stitching images are very different, which directly affects the accuracy, consistency, and repeatability of tumor bed assessment.

When using the software-assisted splicing method, the pictures spliced and restored by two pathologists are extremely similar (Fig. 3b and d), and the measured values are more consistent, which improves the accuracy of tumor bed evaluation after breast cancer neoadjuvant treatment.

### Comparison of the time required for conventional and software-assisted methods to evaluate the tumor bed of specimens

Five pathologists respectively used conventional evaluation methods and “WSI Stitcher” assisted evaluation methods to evaluate the tumor beds of 80 breast cancer specimens after neoadjuvant treatment. The results showed that the time for each doctor to use the software-assisted evaluation was significantly shorter than that of conventional evaluation methods (*P* < 0.001) (Table 1).

**Table 1:** Comparison of the time of each pathologist using conventional assessment and software-assisted assessment of the tumor bed after neoadjuvant chemotherapy

Pathologist	Conventional(M ± SD)(s)	Software(M ± SD)(s)	*P*-value
A	365.9 ± 142.8	238.5 ± 70.2	<0.001
B	369.6 ± 144.9	240.3 ± 75.6	<0.001
C	385.5 ± 159.4	248.9 ± 83.5	<0.001
D	413.5 ± 137.4	217.4 ± 70.0	<0.001
E	449.4 ± 140.2	210.6 ± 71.7	<0.001

**Fig. 4:**
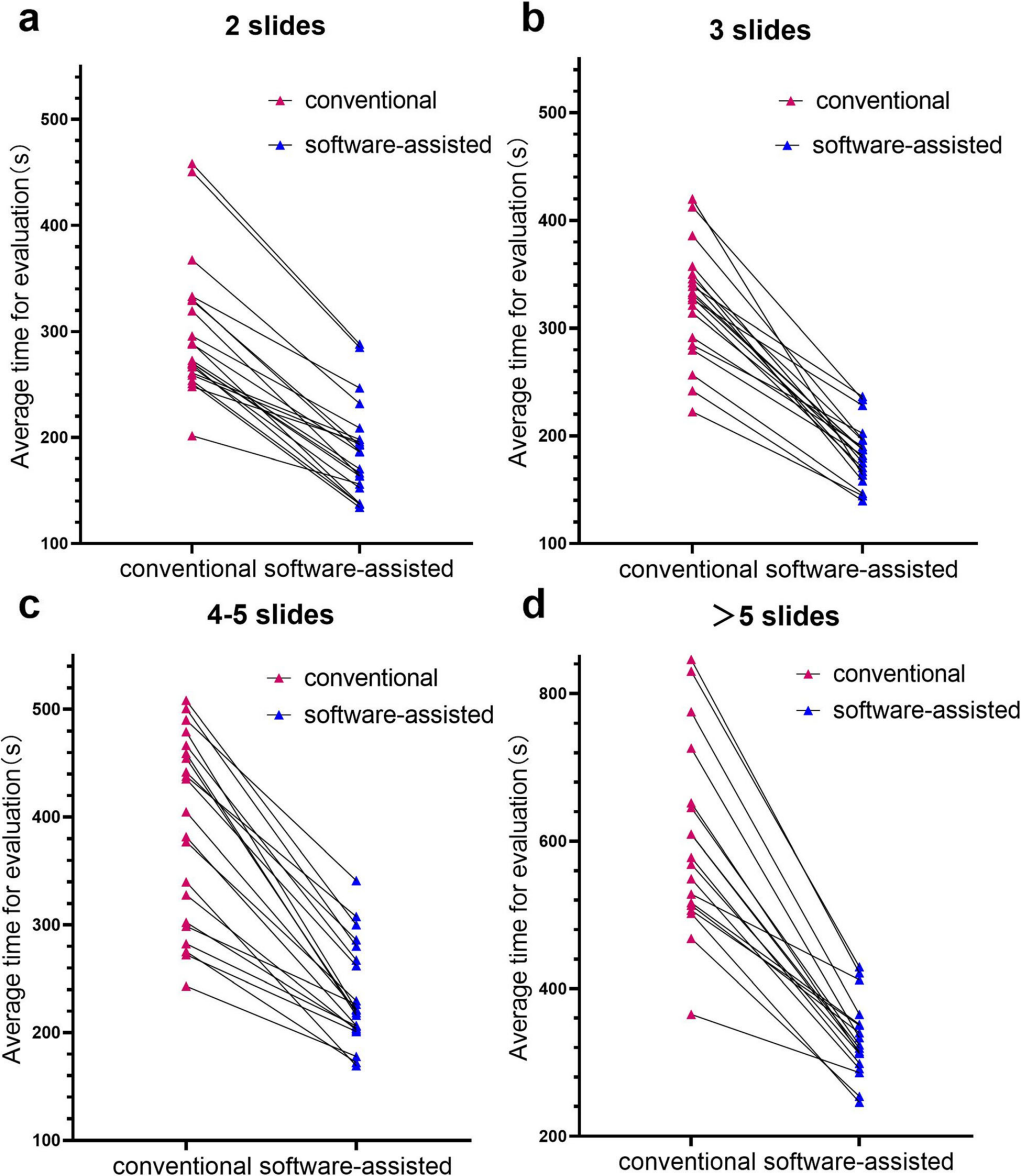
Comparison of the average stitching time using conventional and software-assisted assessment of breast tumor bed. **a**, **b**, **c**, **d** respectively show that the number of slides on the largest section of the tumor bed after neoadjuvant chemotherapy is 2, 3, 4–5, and > 5

We divided 80 cases into 4 groups according to the total number of slides for the largest section of the tumor bed, which were 2 (21/80), 3 (20/80), 4–5 (21/80),> 5 slides (18/80). For these four groups of cases, we drew a line graph of the average time required for interpretation by 5 pathologists using conventional and software-assisted evaluation methods (Fig. 4a-d). The line graph results show that for the 4 groups of cases, the software-assisted evaluation requires significantly less time than conventional methods. For the cases where the largest section of the tumor bed has 2, 3, 4–5 and > 5 tissue fragments, the interpretation time decreased by 36.8 ± 13.3, 42.6 ± 9.2, 39.1 ± 10.1, and 44.6% ± 4.9%, respectively. We found that the WSI-stitcher can save more time as the slide number increases.

### Comparison of consistency between conventional and software-assisted assessment methods for tumor bed evaluation

For 43 cases of residual tumor bed without ductal carcinoma in situ(DCIS), 5 pathologists all put 0 in the tumor bed measurement in both the conventional evaluation method and software-assisted evaluation. Therefore, we excluded these cases and analyzed the consistency of the percentage of DCIS in the remaining 37 cases.

**Fig. 5:**
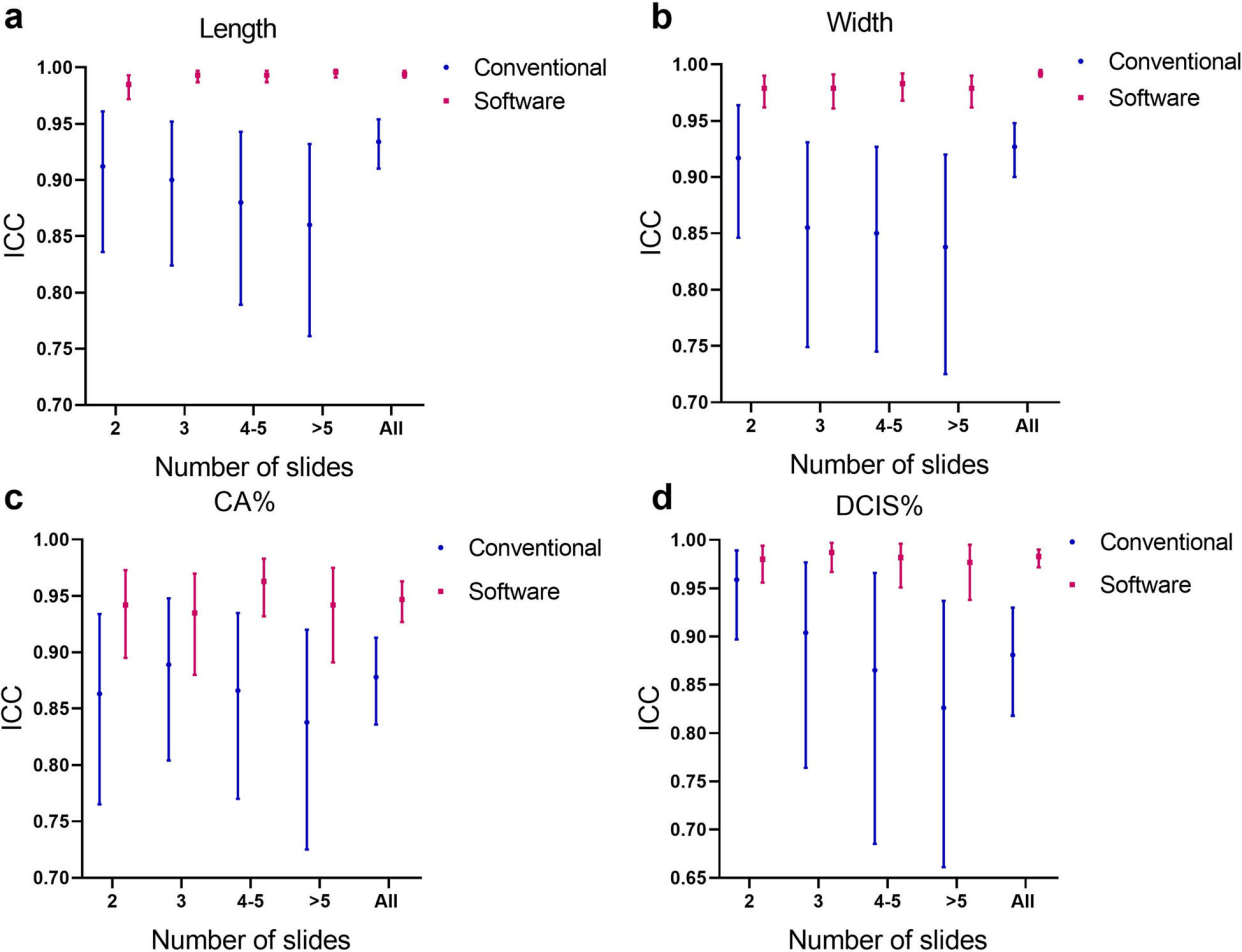
Comparison of tumor bed evaluation consistency between the software-assisted and conventional method by five pathologists. **a-d** Comparison of the consistency between the conventional methods and software-assisted methods in evaluating tumor bed length diameter, width diameter, percentage of tumor cells in tumor bed (CA%) and percentage of ductal carcinoma in situ (DCIS%). ICC: Intraclass correlation coefficient

Compared with the conventional evaluation method, when the number of slides was 2, the ICC of pathologists using the software-assisted method to evaluate the length diameter, width diameter, percentage of tumor cells and percentage of carcinoma in situ increased by 8.0, 6.8, 9.2 and 2.2%, respectively. When the number of slides was 3, the ICC of pathologists using the software-assisted method to evaluate the length diameter, width diameter, percentage of tumor cells and percentage of carcinoma in situ increased by 10.3, 14.5, 5.2 and 9.2%, respectively. When the number of slides was 4–5, the ICC of pathologists using software-assisted method to evaluate the length diameter, width diameter, percentage of tumor cells and percentage of carcinoma in situ increased by 12.8, 15.6, 11.2 and 11.7% respectively. When the number of slides was more than 5, the ICC of pathologists using software-assisted method to evaluate the length diameter, width diameter, percentage of tumor cells and percentage of carcinoma in situ increased by 15.8, 16.8, 12.4 and 18.3%, respectively(Fig. 5a-d).

**Fig. 6:**
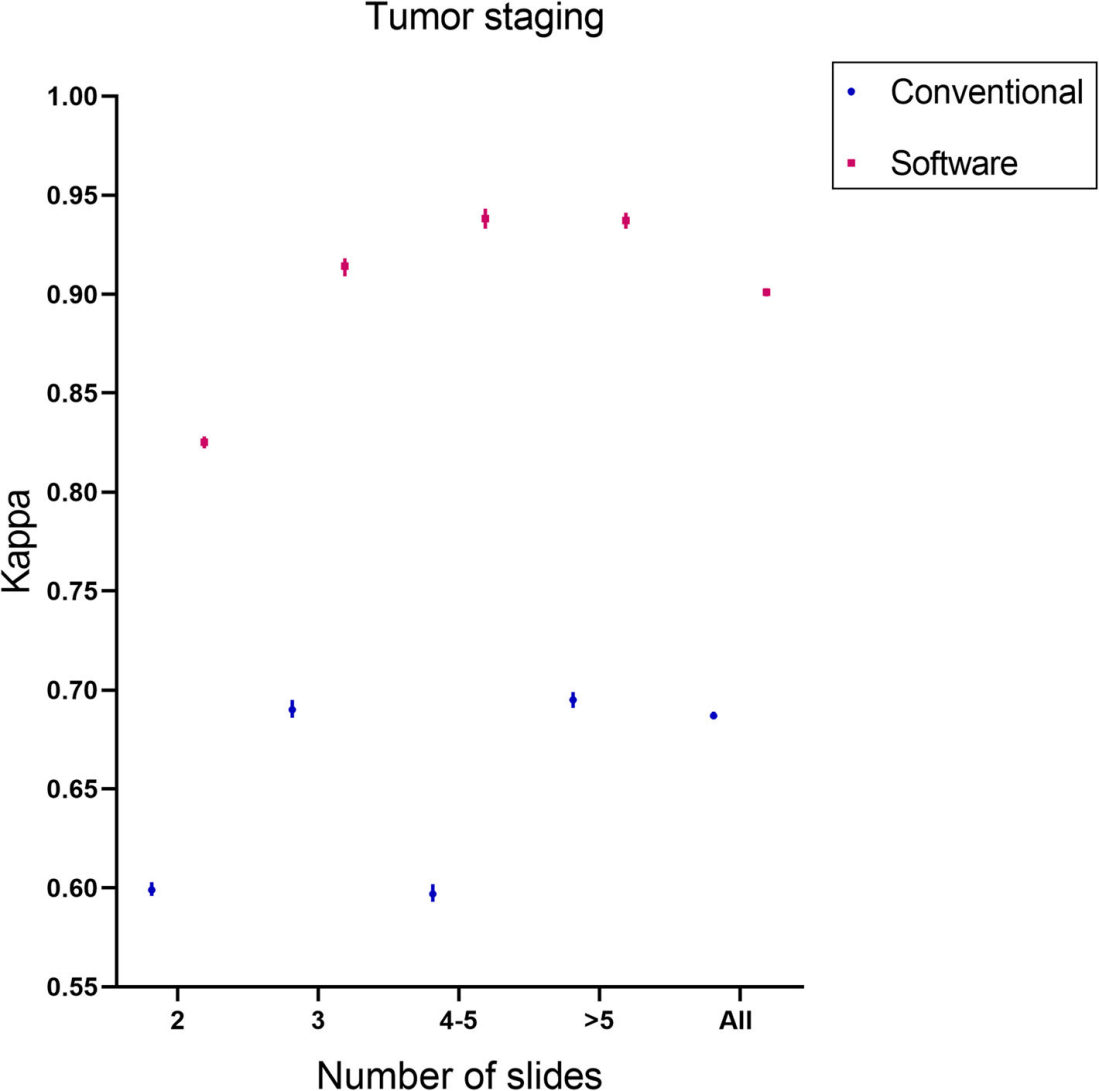
Comparison of tumor staging consistency between the software-assisted method and conventional method by five pathologists

**Fig. 7:**
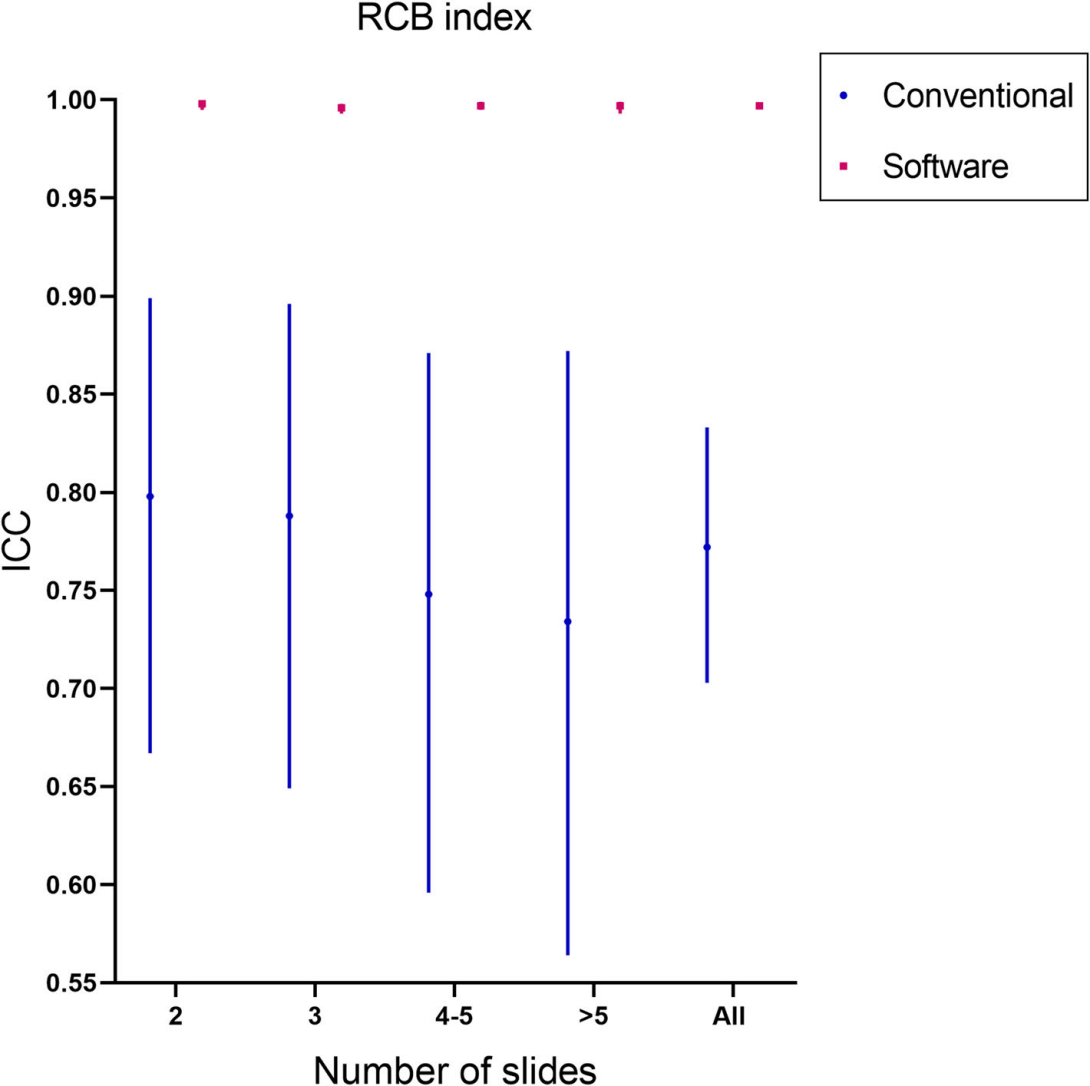
Comparison of RCB index consistency between the software-assisted method and conventional method by five pathologists

**Fig. 8:**
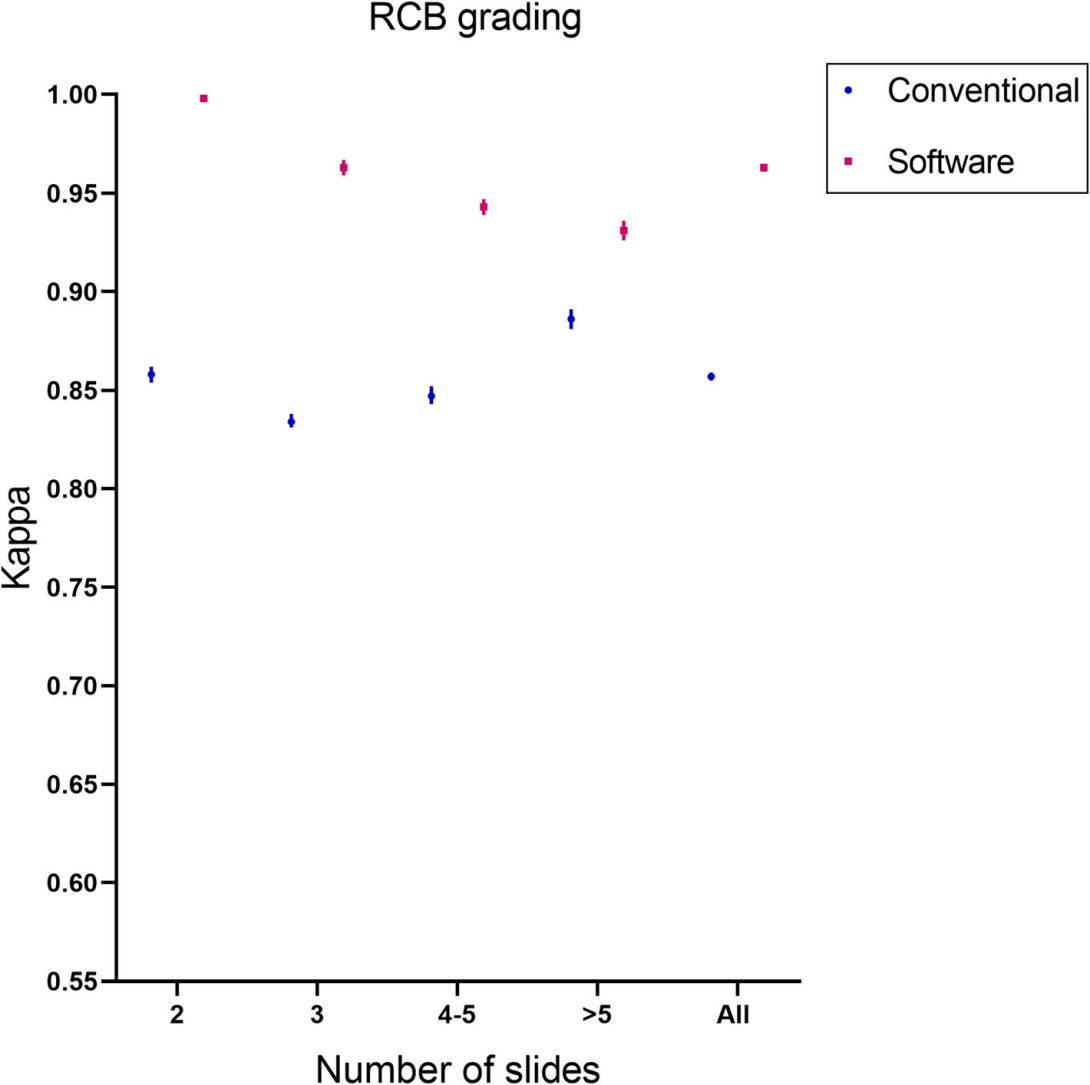
Comparison of RCB grading consistency between the software-assisted method and conventional method by five pathologists

Nine of the 80 patients with involvement of the skin or chest wall were classified as T4 stage, and these cases were excluded. A total of 71 patients were analyzed for tumor staging results. Compared with conventional evaluation methods, the consistency of pathologists using software assisted methods to evaluate tumor staging was improved by 31.1%(Fig.6).Compared with the conventional evaluation method, the ICC of pathologists using software assisted method to evaluate RCB index increased by 29.1% (Fig. 7), and the consistency of evaluating RCB grading increased by 12.4% (Fig. 8).

Pathologists used WSI Stitcher to assist in evaluating the tumor bed after neoadjuvant treatment of breast cancer, which significantly improved the consistency of the long diameter, wide diameter, percentage of residual tumor cells, and percentage of carcinoma in situ. Thus, the consistency of breast cancer tumor staging, RCB index and RCB grading after neoadjuvant therapy was significantly improved.

## Discussion

Tumor bed evaluation in post-NAT breast cancer often requires stacking and assembling multiple slides to measure the maximum diameter of the tumor bed. However, this is an inconvenient process, and holding the stack stable is difficult when there are too many slides involved. Furthermore, each physician may have different stacking order, which will not only lead to differences in the final stitched image but also different measurements for the length and width of the tumor bed, ultimately affecting the accuracy of the final RCB index and T staging. Therefore, pathologists have made several attempts at solving this problem. On one hand, large-section histopathology (LSH) produces large sections (7.5 cm × 5 cm × 5 μm), which allows the observation the entire excised tissue without the tissue fragments stitching process. The LSH was first reported in 1994 by Jackson et al. [22], and subsequently applied to esophageal cancer [23], liver cancer [24], breast cancer [25], prostate cancer [26], and so on. However, since the LSH has a size limitation of 7.5 cm by 5 cm, it is not suitable for larger specimens. On the other hand, the promotion of LSH is limited by a variety of reasons such as the high level of operational difficulty, long cycle of slide preparation, complicated procedures, high costs, and poor slide quality.

On the other hand, WSI fragments stitching programs are developed for the reconstruction of VLS [12–14]. We also independently developed a stitching software for pathological tissue fragments – the WSI Stitcher. It allows the import, rotation, flipping and panning of multiple WSI images to reconstruct gross specimens. This software enables rapid and convenient reconstruction and measurement of VLS and saves the high cost and the tedious work of dehydrating, sectioning and staining required by LSH. Moreover, WSI stitcher can help pathologists to get more accurate pathological measurements, thus facilitating RCB grading for the evaluation of NAT outcomes, and providing guidance for the formulation of subsequent treatment plans.

Our study indicates that compared with the conventional method, the time required for all pathologists to perform tumor bed evaluation in post-NAT breast cancer with the assistance of WSI Stitcher was significantly shorter (*P* < 0.001)(Fig. 4). In the conventional evaluation method, the stacking of more slides makes the tissue area easier obscured by the slide label(Fig. 3a,c). Therefore, pathologists need to try repeatedly before finding a suitable placement and stacking position. This process is very time-consuming. In contrast, with WSI stitcher, pathologists can quickly restore and stitch WSI fragments by referring the cut record on X-ray.

In the tumor bed evaluation process, when using the conventional evaluation method to determine the length and width of the tumor bed, the percentage of residual tumor cells and the percentage of DCIS, the ICC values of five pathologists were 0.934, 0.927, 0.878 and 0.881, respectively. When performing WSI Stitcher-assisted evaluation, the ICC values were 0.994, 0.992, 0.947 and 0.983, respectively. As the number of slides increases, the ICC of the conventional method is declining, indicating that the stitching and measurement difficulty increases with the slide amount. The software-assisted splicing method overcomes this difficulty and remains high tumor bed evaluation consistency as slide amount increases(Fig. 5a-d). We believe the higher ICC is caused by two main reasons. First, in the stitching phases, as we can find in Fig. 3, the final stitching result of conventional methods can be different for everyone. However, with the help of WSI stitcher, the stitching results are almost the same. In the measuring phase, WSI stitcher provides pixel-level measurement accuracy. In contrast, the conventional approach using a ruler for measurements on the stack slides is very rough.

Furthermore, the WSI stitcher can automatically display the percentage of residual tumor cells and the percentage of DCIS once the tumor margin is labeled. These features can effectively enhance the accuracy and efficiency of tumor bed evaluation in post-NAT breast cancer. Therefore, WSI Stitcher exhibited significant advantages in tumor bed evaluation. Compared with conventional evaluation methods, the consistency of pathologists using software assisted methods to evaluate tumor stage, RCB index and RCB grading was significantly improved (*p*<0.001).

Based on clinical experience, we believe that the gold standard for tumor bed evaluation in post-NAT breast cancer is the complete tumor bed obtained using a large-section microtome. Since the complete tumor bed is preserved, there is no need for stitching, nor are there issues of interference from gaps or alignment, and hence this approach should be considered the gold standard that is closest to the true state of the specimen. However, due to time and manpower constraints, we were unable to conduct further research based on the gold standard. Nevertheless, given the various advantages and practicalities of software-assisted stitching, we also believe WSI Stitcher-assisted stitching is an evaluation method that is closer to the gold standard than conventional manual stitching. Thus, it can effectively enhance the accuracy of tumor bed evaluation in post-NAT breast cancer.

In addition, the saved high-quality slides can be used to for future analysis, for example, artificial intelligence-assisted interpretation of indicators such as TILs. Investigations have shown that the tumor regression grade of post-NAT breast cancer [27] is significantly correlated with prognosis, and the emergence of large sections can assist physicians in better evaluating these crucial pathological features. Furthermore, the construction of a WSI database for the patient’s tumor bed can facilitate the easy extraction of saved high-quality large sections of the tumor bed, which can be compared with H&E slides from preoperative needle biopsy, and possible recurrent or metastatic lesions in the future. This will prevent inconsistencies in the tumor bed caused by multiple rounds of manual overlay and stitching. There are broad application prospects for the construction of a tumor bed WSI database in post-NAT breast cancer. It will lay a solid foundation for further in-depth examinations on the pathological morphology of the tumor bed in post-NAT breast cancer, while the preservation of patient information will substantially facilitate repeat examinations of pathological features and future scientific research. This will be of great significance to the patient’s personalized precision treatment and long-term follow-up.

## Conclusion

Based on a large-scale trial involving five pathologists and 80 cases, the “WSI Stitcher”-assisted interpretation is more efficient and consistent than conventional evaluation methods. The advantage of the WSI stitcher-assisted method is more obvious when the amount of slides is large. The high-quality saved virtual large slides can also be used for a variety of future analysis to achieve a more comprehensive assessment of the tumor bed of the specimen after neoadjuvant treatment of breast cancer.

## Data Availability

All data generated or analyzed during this study are included in this published article.

## Abbreviations

NAT: Neoadjuvant therapy
RCB: Residual Cancer Burden
WSI: Whole slide imaging
VLS: Virtual large slides
TILs: Tumor-infiltrating lymphocytes
CXS: Cabinet X-ray system
H&E: Hematoxylin-eosin;
CIS: Carcinoma in situ
DCIS: Ductal carcinoma in situ
ICC: Intraclass correlation coefficient
LSH: Large-section histopathology
